# A comprehensive dataset of mandarin leaf images for classification

**DOI:** 10.1016/j.dib.2025.111685

**Published:** 2025-05-19

**Authors:** Mushfiqur Rahman, Imtiaz Ahmed, Mahin Ahmed

**Affiliations:** Department of Computer Science and Engineering, Daffodil International University, Dhaka, Bangladesh

**Keywords:** Mandarin, Leaf, Classification, Nagpuri, Darjeeling, China mishit, Meandering

## Abstract

The research is devoted to mandarin leaf classification using a deep learning approach. Citrus cultivation has a big market in Bangladesh. In citrus, mandarin is one of them. For the research, we collected images from the Natore district in Bangladesh. The datasets are classifying with the Mandarin varieties are cultivated in Bangladesh. Image classification has been done with Mandarin veritas, such as China Mishti, Nagpuri, Darjeeling, and Meandering. The images were collected in 2608 × 4624 pixels from Realme 8. The raw images are in four varieties, and the total image count is 1917. The image is also attached with augmented images into different augmented sub-folders with a count of eight thousand images. The augmented images are included with image rotations and contrast enhancement. The augmented image count is 8000. All the collected and augmented images are included with healthy images, which help to classify mandarin leaves using the deep-learning approach with reasonable accuracy. The datasets are open for the researchers for their innovative research to advance the leaf classification technique and contribute their research and development to the agriculture domain for enhancing the classification technique with an automated system. The dataset comprises only healthy leaf images, which not only aids in the accurate classification of mandarin leaves using deep learning techniques but also supports early identification of healthy foliage. This is crucial for gardeners and farmers, as it can help detect issues such as mis-grafting in trees, enabling timely interventions.

Specifications Table

**Table:** 

Subject	Computer Science, Agriculture Science
Specific subject area	Image Processing, Leaf Classification, Deep learning
Data format	Raw Data
Type of data	The raw data comprises JPG format photos with RGB colour and a resolution of 2608 × 4624 pixels. And after image processing we convert it 224*224 pixels. All the images are in JPG format.
Data collection	To obtain this dataset, images were gathered from several nursery gardens established in nature. One thousand nine hundred seventeen (1917) raw images are collected from the field were the Mandarin is cultivated. And after pre-process the raw images there have two (2) thousand images in every variety and the total count is eight (8) thousand augmented images.
Data source location	The following nursery fields in Bangladesh are used for data collection:1. Kohinoor Nursery and its sub brunches in Natore district. Date: 28 December 2023 -1 January 2024
Data accessibility	Repository name: Mendeley Dataset Data identification number: doi:10.17632/8bvv2pr2d3.1 Direct URL to data: https://data.mendeley.com/datasets/8bvv2pr2d3/1

## 1 Value of the Data

The Citrus genus belongs to the Rutaceae family and includes various economically significant fruit-bearing trees and shrubs known for their segmented, aromatic fruits. Major species within this genus include:

• Citrus sinensis (sweet orange).
• Citrus limon (lemon).
• Citrus paradisi (grapefruit).
• Citrus aurantiifolia (lime).
• Citrus reticulata (mandarin).

Citrus reticulata, or Mandarin, is a foundational species within the genus, widely recognized as an ancestor to many other citrus varieties and hybrids due to its unique genetic characteristics and adaptability. It is distinguished by its smaller fruit size, thin, easily peelable rind, and sweet, mild flavor. Citrus classification is challenging because of frequent hybridization and high genetic diversity, resulting in species' overlapping morphological and genetic features. Understanding this complex classification framework is essential for researching specific traits, like leaf morphology, as it helps differentiate between closely related Citrus varieties.

The datasets were collected for the mandarin leaf classification, we use a deep-learning approach for develop the classification accuracy. It is essential to deep-learning applications. We collect the data sets for following reasons:

• The datasets were classified with all healthy leaves of four mandarin varieties those cultivate in Bangladesh. It can be useful for researchers working on leaf spot diseases and serve as motivation for further research [1] they can easily classify the healthy leaves.
• The datasets are all are in raw format that help to extract the feature of mandarin four varieties.
• A plant pathologist has authenticated the data, which signifies its credibility and reliability. The Datasets are authenticated by plant pathologists which contain the statement for data authentication
• The datasets also collaborate with augmented images in different folders that help to increase the accuracy of classification results. The datasets are rotated in multiple dimensions that help to increase the classification accuracy.

**Potential Areas for Improvement:**

The contributions of these datasets are collected for agricultural automation development using deep-learning approaches. The data will be more valuable when the user of the datasets will be the researchers who engage themselves resource for future investigations, laying the foundation for crucial developments in these domains [2].

## 2 Background

Due to favourable climatic conditions in Bangladesh, citrus fruits, especially oranges, limes, and lemons, are cultivated in various regions, primarily in Sylhet, Chittagong, and the hill tracts. Citrus cultivation contributes to local economies and provides essential nutrients, particularly vitamin C. However, production faces diseases, inconsistent weather patterns, and limited modern agricultural practices. Efforts are ongoing to enhance yield and quality through improved farming techniques and pest management. In different climates with advanced agricultural techniques, leaf variations are being studied more. In India and China, Big Data technology has highlighted the relevance of primary datasets in agricultural quality and production [3]. With its abundant soil and supportive climate, Bangladesh is a great place to research plants like the Mandarin tree, which has economic and nutritional advantages.

We needed this information to better understand Bangladesh's Mandarin tree botanical variety for study, conservation, and cultivation. In machine learning, datasets are crucial. Computational prediction methods are limited by the lack of standardized and publicly accessible agricultural statistics [4]. Researchers, agriculturists, and governments may use this dataset to fill this gap. Based on the research of seventeen different common for their contain nutrients: Carbohydrate, protein, lipids, vitamins etc. The highest contents of B-Carotene fruits are orange and tomato [5].

One thousand and five hundred hyper spectral camera images of thirty distinct plant species were used to test the research model. The study showed that LtCNN could correctly identify leaf features in photos of living plants with crowns, even though it needed less training data than CNN models with more complex features, like AlexNet, GoogLeNet, and VGGNet. For simulated RGB images, the model's kappa coefficient was approximately 0.90, while for 3-band RGB and near-infrared images, it was 0.95. Huixian (2020) used deep learning and artificial neural networks to classify and identify plant leaf pictures [6].

Due to its geography and climate, Bangladesh has a diversified agricultural environment. Mandarin tree leaf variations are little documented despite their commercial significance. Despite increased support for sustainable agriculture and safeguarding biodiversity, this dataset addresses this gap. It uses botanical and agricultural research to study plant taxonomy, utilizing Mandarin plant leaf pictures from around Bangladesh with plant pathologists verifying correctness.

## 3 Data Description

All images are collected from the nursery, as shown in Table 1. The data is gathered. Four groups of Mandarin varieties are represented in this data set. They are Nagpuri, China Mishti, Darjeeling and Meandering. The datasets are collected from the Natore, Bangladesh in December. We can classify the leaves based on their structure. This could enable rapid quality assessment and decision-making in various industries, such as agriculture, food preservation, and pharmaceuticals [7]. Each quantity of dataset is described in detail below (Table 2):

**Table 1:** Dataset Description.

Category	Number of Images
Nagpuri	366
China Mishti	511
Darjeeling	500
Meandering	540

**Table 2:** Describe the Mandarin leaves verities.

## 4 Experimental Design, Materials and Methods

The dataset represents various mandarin varieties, which are Nagpuri, China Mishti, Darjeeling, and Meandering [3]. The experimental strategy and methods of this work included data collection, dataset preparation, data pre-processing, and data augmentation for better classification accuracy. Including this strategy, we can create a machine learning or deep learning-based application. Because based on the machine learning is rapidly expanding across many industries, including agriculture and the IT sector [8]. The process is described below:

**Data Acquisition:** Images were captured in a nursery from 5- to 6-month-old trees using a high-resolution 64 MP Smartphone camera. A pathologist authenticated the images by identifying and confirming the affected leaves.

**Dataset Preparation:** We divided the dataset based on Mandarin varieties, which include Nagpuri, China Mishti, Darjeeling, and Meandering. Each variety's data is stored in separate folders, labelled with the variety name and the corresponding count.

**Fig. 1:**
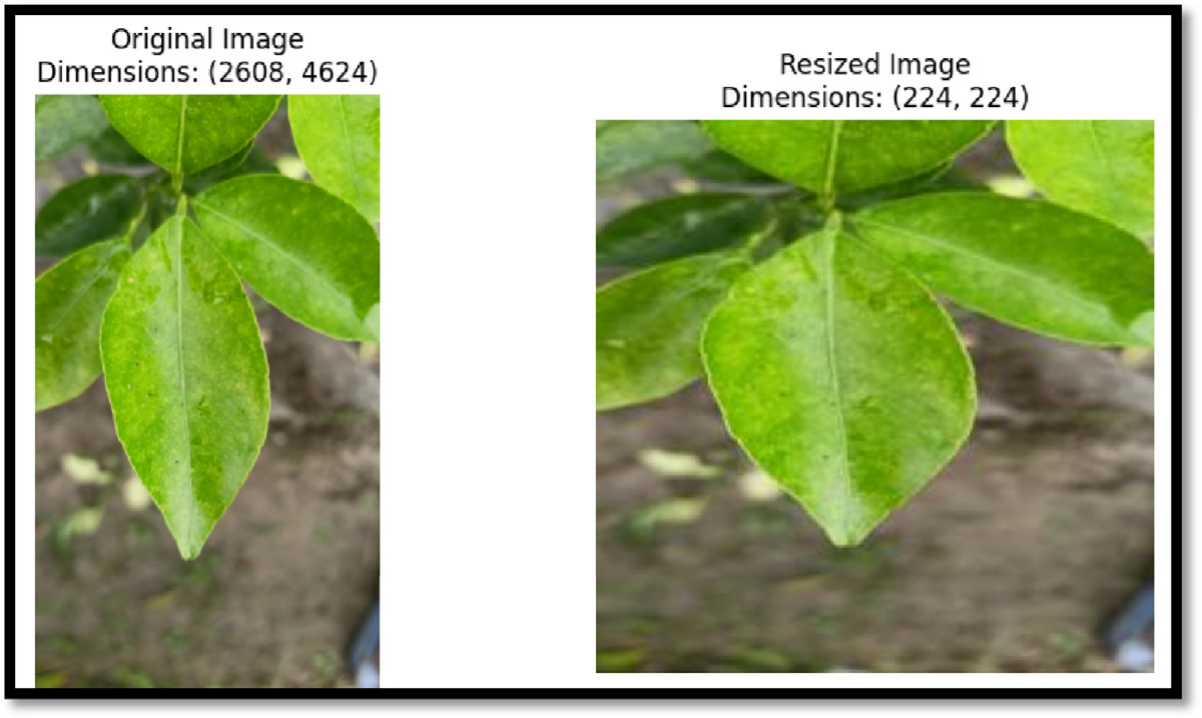
Resized Mandarin Leaf.

**Data Pre-processing:** The data sets was pre-process for the good classification accuracy into different device images and weather. In image pre-processing technique we convert the resolution into 224*224 pixels which is a standard image ratio. In pre-process technique. We use contrast enhancing for extract the feature. The snippet utilizes OpenCV to read an image from a specified file path. It extracts the image dimensions by accessing the shape attribute, which returns height, width, and the number of colour channels. The height and width are stored in separate variables for clarity. Finally, the image dimensions are printed in a formatted string. This process is essential for understanding the input data characteristics prior to pre-processing or analysis in the context of deep learning applications (Fig. 1).

**Fig. 2:**
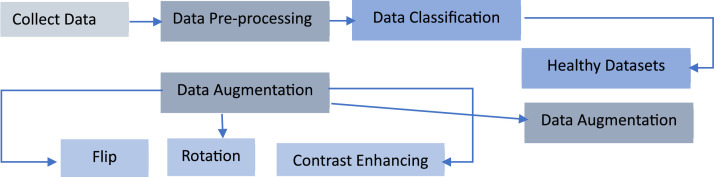
Data Processing Methodology.

**Fig. 3:**
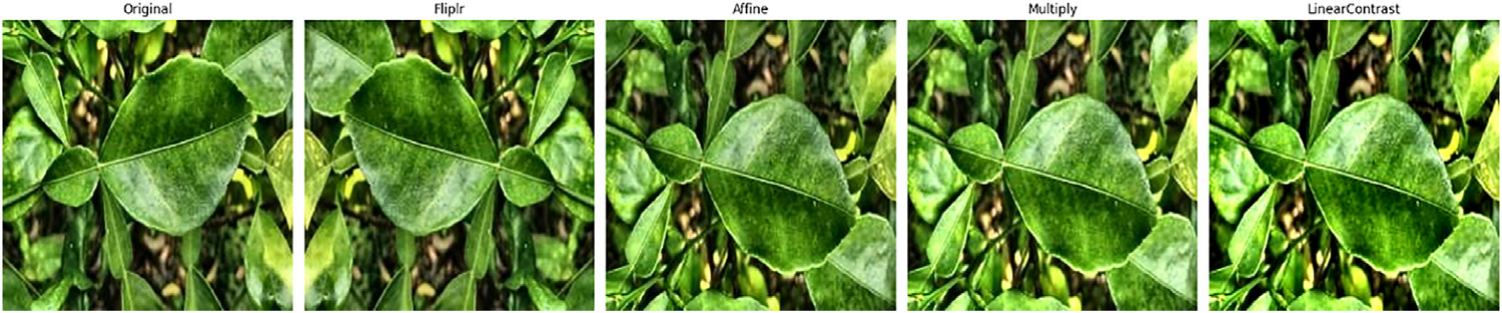
Data Augmentation.

**Data Augmentation:** In mandarin leaf classification data technique place a vital role in improve the accuracy. We try to collect the images in same camera angle for classification. Using augmentation technique, we enhance the data sets into different angles. By use of this dataset, various machine learning techniques can be applied, including deep learning, feature extraction, and pattern recognition, to enhance the accuracy and efficiency of automated sugarcane disease identification systems [2] (Fig. 2, Fig. 3).

Table 3 presents four image categories: Nagpuri (366 images), China Mishti (511 images), Darjeeling (500 images), and Meandering (540 images). To address class imbalance and improve model training effectiveness, each category was augmented to a total of 2000 images. The original and augmented datasets are referenced in [7].

**Table 3:** After augmentation count.

Category	Number of Images	Augmented Count
Nagpuri	366	2000
China Mishti	511	2000
Darjeeling	500	2000
Meandering	540	2000

## Limitations

The datasets are collected for mandarin leaf varieties classification. There have some few limitations on leaf image datasets. The images datasets are organized with healthy leaves. Verities of mandarin leaf images can differ based on light intensity. Secondly the raw datasets size may decreases the accuracy result.

## Ethics Statement

The paper is a unique work by the author and has not been previously published the article is currently not being reviewed for publication anywhere and the datasets are accessible for research purpose to everyone.

## CRediT Author Statement

**Imtiaz Ahmed & Mahin Ahmed**: Methodology **Mr. Mushfiqur Rahman**: Supervision **Dr. Sayed Mohammad Mohsin**: Data Authentication.

## Data Availability

Mendeley DataComprehensive Dataset of Mandarin Leaf Varieties. (Original data).

## References

[1] Jadhav R., Suryawanshi Y., Bedmutha Y., Patil K., Chumchu P. (2023). Mint leaves: dried, fresh, and spoiled dataset for condition analysis and machine learning applications. Data Brief.

[2] Thite S., Suryawanshi Y., Patil K., Chumchu P. (2024). Sugarcane leaf dataset: a dataset for disease detection and classification for machine learning applications. Data Brief.

[3] Arman S.E., Bhuiyan M.A.B., Abdullah H.M., Islam S., Chowdhury T.T., Hossain M.A. (2023). BananaLSD: a banana leaf images dataset for classification of banana leaf diseases using machine learning. Data Brief.

[4] Islam S. (2023). BDMediLeaves: a leaf images dataset for Bangladeshi medicinal plants identification. Data Brief.

[5] Bazán-Vera W., Bermeo-Almeida O., Cardenas-Rodriguez M., Ferruzola-Gómez E. (2019). A brief review of big data in the agriculture domain. Commun. Comput. Inf. Sci..

[6] D. P. Hughes and M. Salathe, “An open access repository of images on plant health to enable the development of mobile disease diagnostics,” arXiv.org, 2015. https://arxiv.org/abs/1511.08060

[7] Ahmed I. (2024). Comprehensive dataset of mandarin leaf varieties. Mendeley Data.

[8] Khan M.A.H., Rahim M.A., Robbani M., Hassan M.F., Haque M.A., Alam Z. (2021). Physical and chemical properties of sweet orange genotypes available in Bangladesh. Plant Arch..

